# Supplementation of Non-Starch Polysaccharide Enzymes Cocktail in a Corn-Miscellaneous Meal Diet Improves Nutrient Digestibility and Reduces Carbon Dioxide Emissions in Finishing Pigs

**DOI:** 10.3390/ani10020232

**Published:** 2020-02-01

**Authors:** Yuxia Chen, Dan Shen, Lilan Zhang, Ruqing Zhong, Zhengqun Liu, Lei Liu, Liang Chen, Hongfu Zhang

**Affiliations:** 1 State Key Laboratory of Animal Nutrition, Institute of Animal Science, Chinese Academy of Agricultural Sciences, Beijing 100193, China; yuxiatianya0428@126.com (Y.C.); 18603723064@163.com (D.S.); zhanglilan92@163.com (L.Z.); ruqing_zhong@163.com (R.Z.); liuzhengqun2015@163.com (Z.L.); swina2010@163.com (L.L.); 2 Precision Livestock and Nutrition Unit, Gembloux Agro-Bio Tech, TERRA Teaching and Research Centre, Liège University, Passage des Déportés 2, 5030 Gembloux, Belgium

**Keywords:** non-starch polysaccharide enzymes cocktail, growth performance, nutrient digestibility, carbon dioxide emissions, ammonia, finishing pigs

## Abstract

**Simple Summary:**

The objective of the present study was to investigate the effect of the non-starch polysaccharide enzymes cocktail (NSPEC) on growth performance, nutrient digestion and gas emissions on finishing pigs. The addition of the NSPEC into a corn-miscellaneous meal diet improved feed conversion ratio and increased the apparent total tract digestibility of dry matter, neutral detergent fiber, acid detergent fiber, crude protein and gross energy of the finishing pigs. Furthermore, the digestible energy of the diet was also increased by the NSPEC supplementation in the diet. In addition, the inclusion of NSPEC in the corn-miscellaneous meal diet reduced carbon dioxide emissions of a finishing pig house. The accumulation of manure could increase the release of ammonia in a pig house.

**Abstract:**

This study was carried out to evaluate the effect of the addition of the non-starch polysaccharide enzymes cocktail (NSPEC) on growth performance, nutrient digestibility and gas emissions in a corn-miscellaneous meal-based diet for finishing pigs. The NSPEC is a combination of cellulase, xylanase, β-glucanase, β-mannanase, α-galactosidase and pectinase optimized by assessing the in vitro dry matter digestibility (IVDMD) of corn-miscellaneous meal diet using an in vitro method of simulating digestion in the stomach and intestine of growing pigs. Growth performance and apparent total tract digestibility (ATTD) of nutrients and energy were measured. The gas concentration of ammonia, carbon dioxide, nitrous oxide and methane in the environmental assessment chambers were determined. The gas detecting period was divided into three frequencies of manure removal of every 1d, 2d and 3d. The addition of NSPEC into the corn-miscellaneous meal diet decreased feed conversation rate (FCR) and increased the ATTD of dry matter, crude protein, gross energy, neutral detergent fiber and acid detergent fiber of pigs (*p* < 0.05). The digestible energy was also improved (*p* < 0.05) significantly by NSPEC supplementation in the diet. Furthermore, the supplementation of the NSPEC reduced (*p* < 0.05) carbon dioxide concentration in the chambers. The ammonia emissions were significantly increased according to average 1d, 2d and 3d manure removal procedures (*p* < 0.01). These results indicated that the inclusion of optimal NSPEC in a corn-miscellaneous meal diet improved growth performance, nutrient digestibility and reduced carbon dioxide emissions on finishing pigs. The accumulated manure could increase the release of ammonia in a pig house.

## 1. Introduction

Many feed ingredients contain non-starch polysaccharides (NSP), which act as anti-nutritional substances. These NSP cannot be broken down by endogenous enzymes, which will encapsulate other nutrients, increase the endogenous nutrient loss, and then result in lower nutrient and energy digestibility [1,2]. Therefore, it is necessary to supplement the appropriate NSP enzyme, especially NSP enzyme cocktail (NSPEC), in a variety of feeds to degrade the anti-nutrient factor and improve growth performance of animals. Previous studies showed that the supplementation of NSP enzyme complex (β-glucanase and xylanase) improved average daily gain of weaned and growing pigs [3,4]. Our early research demonstrated that the optimized enzyme cocktail obtained by in vitro method of corn-soybean and corn-miscellaneous meal diet could more effectively improve the in vitro nutrient digestibility [5]. 

Some studies showed that the supplementation of enzymes in the diet can reduce harmful gas emissions. Agriculture and livestock husbandry are important contributors to global emissions of greenhouse gases (GHG), namely nitrous oxide (N_2_O), methane (CH_4_) and carbon dioxide (CO_2_). Pig production is the second largest source of GHG emissions in livestock husbandry [6]. Manure management is the primary source of GHG emissions from pig production, which in turn accounts for 18% of the total global GHG emissions from the livestock industry [7]. Moreover, global pig production accounts for approximately 15% of livestock-related ammonia (NH_3_) emissions [8]. The environmental problems resulting from GHG emissions and deposition of NH_3_ from animal respiration and manure has raised global concern. Manipulation of the pigs’ diets could alter the composition of the manure [9]. Some physical and chemical methods had been used to reduce NH_3_ and GHG emissions in the swine industry [10,11,12]. Until now, these methods have not been widely applied in swine production because they are all economically expensive and lack certain stability. It is urgent that new solutions to reduce environmental burdens are found. Therefore, more economic and stable biological methods such as probiotics from anaerobic microflora [13,14], herb extract mixtures [15], acidifying salts [16] and enzyme products are gradually used in the porcine industry [17,18]. Nielsen et al. examined that phytase supplementation in pig diets contributed to improving the environment [19], while the studies by Oxenbøll et al. and Anja et al. found that the supplementation of protease and xylanase, α-amylase and protease cocktail in broiler diets could be beneficial to the environment [20,21].

However, little information was found on the effect of the NSPEC optimized using an in vitro method by assessing the in vitro dry matter digestibility (IVDMD) of corn-miscellaneous meal diet on nutrient digestion and gas emissions for finishing pigs. Therefore, the aim of this study is to evaluate the effect of the optimized NSPEC supplementation in a corn-miscellaneous meal diet on growth performance, nutrient digestibility and gas emissions on finishing pigs.

## 2. Materials and Methods

All procedures were approved by the Institutional Animal Care and Use Ethics Committee in the Institute of Animal Science of the Chinese Academy of Agricultural Sciences and all pig treatments were carried out in accordance with the Regulations for the Administration of Affairs Concerning Experimental Animals of the State Council of the People’s Republic of China (Ethics Approval Code: IAS-2018-4). 

### 2.1. Diets, Animals and Experimental Design

Corn-miscellaneous meal-based diet (CT diet) and CT diet with the addition of NSP enzyme cocktail (NSPEC diet) were prepared in this study. The ingredients and the chemical composition of the test diets are summarized in Table 1. The feed was manufactured in dry mash form and formulated to meet or slightly exceed the nutritional requirements of finishing pig as recommended by the National Research Council [22]. 

The methodology for screening the NSPEC in a diet using an in vitro method of simulating digestion in the stomach and intestine of pigs was developed in our lab [5]. The optimal NSP enzyme cocktail in the corn-miscellaneous meal diet was 1002 U/kg cellulase, 18,076 U/kg xylanase, 1376 U/kg β-glucanase, 14,765 U/kg β-mannanase, 337 U/kg α-galactosidase and 138 U/kg pectinase in the corn-miscellaneous meal diet. The NSPEC was screened by assessing the IVDMD of the corn-miscellaneous meal diet using an in vitro method of simulating digestion in the stomach and intestine of growing pigs. 

Sixteen crossbred barrows (Duroc × (Landrace × Large White)); initial body weight of 117.89 ± 0.85 kg; Beijing Breeding Swine Center, Beijing, China) were randomly allotted into 2 dietary treatments and each treatment had 8 replicates which were randomly divided into 2 environmental control chambers and each chamber had 4 pigs. Each environmental control chamber fed 4 pigs which were housed in stainless steel metabolic cages (1.2 m × 1.5 m). The daily feed allowance was calculated at 3.5% of the initial weight of pigs. Pigs were fed one-half of the daily feed allowance each at 8:00 and 15:00 per day and provided ad libitum access to water during the entire experimental period. After a 5d adaption period and 3 d feces collection period, feces were collected via grab sampling and stored at −20 °C immediately after collection. To avoid differences between the environmental control chambers, the pigs of the CT group and NSPEC group were exchanged between the two experimental periods, and each period had a 3d gas detecting period. The 3d gas detecting period was divided into 3 frequencies of manure removal of every 1d, 2d and 3d. 

### 2.2. Chemical Analysis

At the completion of the experiment, feces samples were thawed, mixed and oven-dried at 65 °C for 96 h. The feed and feces samples were ground through a 0.5 mm sieve in a centrifugal grinder before analysis. All samples of diets and feces were analyzed for dry matter (DM, method 930.15) and crude protein (CP, method 990.03) following the procedures outlined by the Association of Official Analytical Chemists [23]. Neutral detergent fiber (NDF) and acid detergent fiber (ADF) were determined using filter bags and fiber analyzer equipment following a modification of the procedures [24]. Samples of the feed were analyzed for extract ether (EE; method 954.02) and ash (method 942.05) [23]. The gross energy (GE) in the diets and feces were determined using an adiabatic bomb calorimeter (Model 6400; Parr Instrument, Moline, IL). All the analyses were performed in duplicate.

### 2.3. Calculations

The ATTD of DM, CP, NDF, ADF and GE were calculated in the diets according to former reported equations [25]:

ATTD_nutrient_ = [(N_i_ − N_o_)/N_i_] × 100 (1)

where ATTD is the apparent total tract digestibility of gross energy (%), N_i_ is the total intake of DM, CP, NDF, ADF and GE in the feed, and N_o_ is the total fecal output of DM, CP, NDF, ADF and GE.

### 2.4. Measurements of Gas Concentrations

The concentration of gas in all the chambers was measured with a Photoacoustic Field Gas-Monitor INNOVA 1412 (LumaSense Technology, Santa Clara, CA, USA). Simultaneous measurement of NH_3_, N_2_O, CH_4_ and CO_2_ was carried out throughout the entire experiment. The gas emissions expressed per day and per livestock unit were corrected to 500 kg body weight. This system was designed to continuously monitor incoming and exhaust concentrations of gases, including control chamber ventilation volume, temperature and humidity to ensure the stability of the cabin environment [26]. 

A TH100 thermal gas mass flow meter was placed at the ventilator vent to monitor the ventilation environmental controlled chamber in real time. All gas samples were collected in the middle of the intake and exhaust ducts. At the time of gas collection, the gas sampling system continuously delivered the collected gas to the gas detector. Each cabin gas sample was measured 5 times in a sequential manner (every time for 1 min) and it was continuously measured for 24 h.

(2) $$\mathrm{Egas}=D\times \frac{\left(\mathrm{Co}-\mathrm{Ci}\right)}{N}\times T\times \frac{M}{22.4}\times \frac{273}{273+t}\times 60\times 10^{-6}$$

Egas: daily gas emissions of per pig (g/pig/d); 

D: ventilation rate at house temperature and pressure, (L/min); 

Co: concentration of exhaust house ventilation air (ppm); 

Ci: gas concentration of incoming house ventilation air (ppm); 

N: the number of pigs in the house; 

T: ventilation time (24 h);

M: molecular weight of the gas; 

t: the temperature in the house;

Thereafter, the daily emissions were calculated for each series of measurements and expressed per pig and per livestock unit (LU) that equals 500 kg body weight.

### 2.5. Statistical Analysis

The UNIVARIATE procedure (SAS Version 9.2, SAS institute Inc., Cary, NC, USA) was used to confirm the homogeneity of variance and also analyze for outliers, but no outliers were identified. The normality of the data distribution was also tested prior to the final comparison by SAS. Growth performance and nutrient digestibility data were analyzed by Student’s *t*-test. Diet was a categorical variable. Frequency of manure removal was treated as an ordinal variable. According to a completely randomized design for gas emissions, the diet, frequency of manure removal and diet × frequency of manure removal interaction were treated as fixed effects, whereas animals, chambers and periods were treated as random effects by using the MIXED procedure of SAS. The differences were considered significant if *p* < 0.05 and were considered a trend if the *p*-value was between 0.05 and 0.10.

## 3. Results

### 3.1. Growth Performance and Nutrient Digestibility

The supplementation of the NSPEC in corn-miscellaneous meal diet had no effect on the average daily gain (ADG) and feed intake (FI) in finishing pigs (Table 2). However, the pigs fed the NSPEC diet had lower feed conversion rate (FCR) than the CT diet (*p* < 0.05). Compared with the CT group, the inclusion of the NSPEC improved ATTD of DM by 2.4%, CP by 2.84%, NDF by 4.9% and ADF by 5.93% during the overall period (*p* < 0.05). In addition, the ATTD of the GE and DE were also improved by the NSPEC supplementation (*p* < 0.05).

### 3.2. Gas Emission

Table 3 presents the overall patterns of gas emissions including the NH_3_, N_2_O, CH_4_ and CO_2_. The results showed that there was no significant interaction between NSPEC and frequency of manure removal on NH_3_, N_2_O, CH_4_, and CO_2_ emissions. The ADEU (average daily gas emissions per LU) of NH_3_, N_2_O and CH_4_ ranged from 24.30 to 38.21 g, 1.61 to 2.30 g and 10.99 to 14.29 g for fattening pigs according to different frequency of manure removal, respectively. The NH_3_ emissions were significantly increased according to average 1d, 2d and 3d manure removal procedures (*p* < 0.01). The evolution of N_2_O and CH_4_ emissions showed no particular trends throughout the experimental period. No differences were observed for the N_2_O and CH_4_ emissions compared the NSPEC group with the CT group. However, the ADC (average daily gas concentration) of CO_2_ was significantly lower (*p* < 0.05) in the NSPEC group than in the CT group. Furthermore, the ADEU of CO_2_ was remarkably decreased or showed a downward trend (*p* = 0.06) in pigs fed a diet supplement with NSPEC.

## 4. Discussion

In the present study, corn-miscellaneous meal diet with high fiber content was mainly composed of corn, soybean meal, wheat bran, cottonseed meal and sugar beet pulp. The xylan content accounts for the main part of the NSP composition of these feed ingredients. In addition, the mannan content also occupies a large proportion. Therefore, the enzyme cocktail mainly included xylanase and β-mannanase in our study. The pigs fed the NSPEC diet had a relatively lower FCR than the pigs fed the CT diet. The reduced FCR might result from the supplementation of enzyme cocktail in the diet improving the relative NSP digestibility. Our results are also in agreement with a previous study which reported that non-starch polysaccharide-degrading enzymes supplementation improved the FCR of growing pigs fed diets with multi-enzyme [27]. In addition, some studies observed that a complex of non-starch polysaccharide-degrading enzymes could improve the growth performance of the weaned piglets and growing-finishing pigs [28,29,30,31]. However, some studies failed to observe a positive effect of enzyme cocktail supplementation on growth performance of pigs [32]. The apparent contradictions in the effectiveness of multi-enzyme supplementation on growth performance among studies may be mainly attributed to the differences in age of the pigs and the composition of diets used. In addition, the enzyme source and the combination of various NSP enzymes may also exert a different effect on growth performance. 

The NSPEC supplementation increased the ATTD digestibility of DM, NDF and ADF by 2.4%, 4.9% and 5.93%, respectively in growing pigs. The ATTD of CP, GE and DE were also improved when the NSPEC was added in the diet. These results were consistent with a previous study which reported that multi-enzyme supplementation increased nutrient digestibility in pigs [33,34]. For example, Li et al. reported an increase in DM, GE and CP in growing pigs fed a diet supplemented with an amylase, protease and xylanase blend compared to a corn-soybean meal-based diet [35]. The improvement in nutrient digestibility in our study indicated that the NSPEC exerted its beneficial effects on nutrient digestibility of the finishing pigs, probably through first breaking down the plant cell wall structure and then releasing the nutrients for use by the pig [36].

The level of gas concentrations is the balance between the production by the animals’ respiration and/or the manure and the evacuation by the exhaust fans. All the NH_3_, N_2_O, CH_4_ and CO_2_ emission patterns reflect the increase of feed intakes, the higher metabolism of animals and the accumulation of manure during the entire experiment. We found that the NH_3_ emissions were significantly increased according to average 1d, 2d and 3d manure removal procedures. The N_2_O, CH_4_ and CO_2_ emissions showed an upward trend with the accumulation of manure. A previous study has reported that accumulated manure could increase the release of gases by a pig house [37]. Furthermore, manure removal frequency has been proposed to serve as an efficient means to reduce the emissions of harmful gases from pig buildings. Some researchers found that cumulative CH_4_ emissions were shown to be lower by 16% and N_2_O emissions remained the same when manure was removed three times a week instead of only one time in growing pigs [38]. The NH_3_ emissions data demonstrated that there were no significant differences observed between the NSPEC and CT group. The results of this experiment are consistent with a previous study in which exogenous enzyme supplementation in cereals increased ammonia emissions on finisher pigs [39]. However, it was observed that enzyme supplementation decreased ammonia emissions in wheat based diets, while in barley-based diets enzyme supplementation increased ammonia emissions in pigs [40,41]. These opposing results could be elucidated by the difference of the NSP composition of these cereals. For instance, the NSP fraction of barley mainly contains a mixture of β-glucans and arabinoxylans, while arabinoxylans are the main NSP component of the wheat [42]. In pig houses, the formation of nitrous oxide originates only from manure. N_2_O is an intermediate product and its formation mainly takes place during incomplete nitrification and denitrification processes [43]. The supplemental NSPEC in the diets had no significant effect on N_2_O emissions for fattening pigs. There were a few data on N_2_O emissions in the literature, which accounts for approximately 10% of the NH_3_ emission mass [44]. Some authors suggested that reduced NH_3_ emission strategies could also limit N_2_O emissions since NH_3_ is the precursor of the formation of N_2_O [45]. Another greenhouse gas in pig houses is methane. CH_4_ emissions mainly result from enteric fermentation in the gut [46]. In our study, the levels of CH_4_ emissions were not altered after the NSPEC treatment. However, some authors observed a tendency for higher CH_4_ emissions with xylanase supplementation [47]. Although the amount of CH_4_ emissions from pig houses is thought to be very little, its emissions are closely related to the fiber content in the diet [48,49].

The carbon dioxide emissions from pig houses mainly originate from two sources including exhalation by pigs and release from manure. Several authors measured a 25% reduction in CO_2_ emissions from pig breathing, as a consequence of reduced pig activity [50]. CO_2_ release from manure was ignored for many decades [51,52]. However, some researches indicated that the levels of CO_2_ emissions from manure have been evaluated to be 4–5% of the entire amount of CO_2_ exhaled by livestock [53]. The CO_2_ emissions of manure principally comes from three sources, namely: (1) the rapid hydrolysis of urea into NH_3_ and CO_2_ catalyzed by the urease; (2) the aerobic degradation of organic matter; (3) the anaerobic fermentation of organic matter into intermediate product such as volatile fatty acids (VFAs), CH_4_ and CO_2_ [54]. The third process is usually regarded as the principal source of CO_2_ [55]. The present study indicated that the ADC of CO_2_ in the supplemental NSPEC group was significantly lower than the CT group. These results may correspond to the increase of the ATTD of NDF and ADF in the NSPEC supplementation group. We have demonstrated for the first time that supplementation with NSPEC could reduce CO_2_ emission from swine houses according different frequencies of manure removal. Nevertheless, further investigations have to be carried out to clarify detailed gas emission mechanisms under supplemental NSP enzyme cocktail. We only showed that the optimal NSPEC supplementation in corn-miscellaneous meal-based diet could reduce CO_2_ emissions and increase NH_3_ emissions with the manure accumulation for finishing pigs. Therefore, further studies need to be carried out in order to evaluate the effect of the optimal NSPEC on NH_3_ and GHG emissions in fattening pigs fed other diets.

## 5. Conclusions

In conclusion, the supplementation of NSPEC in a corn-miscellaneous meal-based diet could improve pigs’ growth performance. Furthermore, the beneficial effects of the NSPEC supplementation in pigs’ diet on nutrient digestibility improvement and CO_2_ emission reduction are more obvious compared with the CT diet. Therefore, supplemental NSPEC could promote swine production efficiency and improve the feeding environment of fattening pigs. In the meantime, the accumulated manure could increase the release of ammonia in pig houses.

## Figures and Tables

**Table 1:** The ingredient and nutrient composition of experimental diets (as-fed basis).

Items	Diet ^1^	
	CT	NSPEC
Ingredient, %		
Corn	62.00	62.00
Soybean meal	11.00	11.00
Wheat bran	10.00	10.00
Cottonseed meal	5.00	5.00
Sugar beet pulp	8.00	8.00
Limestone	1.20	1.20
Dicalcium phosphate	1.40	1.40
Premix ^2^	1.00	1.00
Salt	0.40	0.40
Nutrient composition ^3^		
DM, %	88.26	88.45
CP, %	16.95	17.20
Ether extract, %	3.62	3.56
Ash, %	6.49	6.45
NDF, %	15.83	15.60
ADF, %	5.49	5.48
Calcium, %	0.75	0.73
Total phosphorus, %	0.52	0.52
GE, MJ/kg	18.42	18.43
Indispensable AAs, %		
Lysine	0.74	0.77
Methionine	0.12	0.14
Threonine	0.33	0.32
Arginine	0.87	0.90
Histidine	0.42	0.39
Isoleucine	0.50	0.49
Leucine	1.32	1.28
Phenylalanine	0.68	0.65
Valine	0.74	0.70
Dispensable AAs, %		
Alanine	0.80	0.82
Aspartate	1.34	1.33
Cystine	0.13	0.16
Glutamic acid	2.84	1.61
Glycine	0.60	0.61
Proline	0.73	0.72
Serine	0.72	0.72
Tyrosine	0.33	0.32

^1^ CT = corn-miscellaneous meal diet; NSPEC = non-starch polysaccharide enzymes cocktail diet. The optimal NSP enzyme cocktail was 1002 U/kg cellulase, 18,076 U/kg xylanase, 1376 U/kg β-glucanase, 14,765 U/kg β-mannanase, 337 U/kg α-galactosidase and 138 U/kg pectinase in the corn-miscellaneous meal diet. ^2^ The premix provided the following per kg of diets: Vitamin A 8250 IU, Vitamin D_3_ 825 IU, Vitamin E 40 IU, Vitamin K_3_ 4 mg, biotin 0.2 mg, chloride 600 mg, folic acid 2 mg, nicotinic acid 35 mg, pantothenic acid 15 mg, Vitamin B_2_ 5 mg, Vitamin B_1_ 1 mg, Vitamin B_6_ 2 mg, Vitamin B_12_ 25 μg, CU as copper sulfate 50 mg, I as potassium iodide 0.5 mg, as ferrous sulfate 80 mg, as manganese sulfate 25 mg, as sodium selenite 0.15 mg, as zinc sulfate 100 mg. ^3^ DM = dry matter; GE = gross energy; CP = crude protein; NDF = neutral detergent fiber; ADF = acid detergent fiber; AA = amino acid.

**Table 2:** Effect of the optimal NSPEC on growth performance and nutrient digestibility in finishing pigs ^1^.

Item	Diet		SEM	*p*-Value
	CT	NSPEC		
Growth performance				
Initial body weight, kg	117.80	117.99	1.662	0.96
Final body weight, kg	136.94	138.89	1.689	0.75
ADG, kg/day	0.87	0.95	0.042	0.36
FI, day	3.34	3.18	0.079	0.35
FCR	3.89	3.42	0.122	0.04
Nutrient digestibility, %				
DM	84.22	86.62	0.494	<0.01
CP	82.38	85.22	1.131	0.02
NDF	70.38	75.28	1.038	0.01
ADF	66.47	72.40	1.235	<0.01
GE	83.93	88.39	1.085	0.03
Digestible energy, MJ/kg	15.38	16.26	0.204	0.02

^1^ DM = dry matter; GE = gross energy; CP = crude protein; NDF = neutral detergent fiber; ADF = acid detergent fiber, ADG = average daily gain; FI = feed intake, FCR = feed conversion rate; CT = corn-miscellaneous meal diet; NSPEC = non-starch polysaccharide enzymes cocktail diet.

**Table 3:** The NH_3_, N_2_O, CH_4_ and CO_2_ emissions from pigs fed a CT diet or diet containing NSPEC with different manure removal frequencies ^1^.

Item	CT			NSPEC			SEM	*p*-Value		
	1d	2d	3d	1d	2d	3d		Removal Frequency	Diet	Interaction
NH_3_										
ADC, g/pig/d	6.18	7.63	9.40	6.21	7.95	10.75	0.52	0.01	0.53	0.82
ADEU, g/pig/d	24.30	30.05	37.00	24.40	31.25	38.21	2.01	0.01	0.52	0.81
N_2_O										
ADC, g/pig/d	0.42	0.48	0.54	0.56	0.54	0.54	0.04	0.64	0.71	0.96
ADEU, g/pig/d	1.61	1.87	2.09	2.18	2.13	2.09	0.17	0.63	0.61	0.96
CH_4_										
ADC, g/pig/d	2.81	3.10	3.46	2.91	3.02	3.60	0.16	0.29	0.66	0.99
ADEU, g/pig/d	10.99	12.13	13.52	11.53	11.16	14.29	0.62	0.28	0.55	0.99
CO_2_										
ADC, kg/pig/d ADEU, kg/pig/d	2.74	2.88	3.00	2.16	2.32	2.50	0.18	0.82	0.04	0.92
	6.36	6.57	6.77	4.95	5.38	5.77	0.48	0.89	0.06	0.99

^1^ 1d = daily manure removal; 2d = every other day manure removal; 3d = every three days manure removal; ADC = average daily gas concentration; ADEU = average daily gas emissions per LU; LU = livestock unit, equal to 500 kg body weight; CT = corn-miscellaneous meal diet; NSPEC = non-starch polysaccharide enzymes cocktail diet. Removal frequency = manure removal frequency; Interaction = removal frequency × diet.
